# Fluctuations, Finite-Size Effects and the Thermodynamic Limit in Computer Simulations: Revisiting the Spatial Block Analysis Method

**DOI:** 10.3390/e20040222

**Published:** 2018-03-24

**Authors:** Maziar Heidari, Kurt Kremer, Raffaello Potestio, Robinson Cortes-Huerto

**Affiliations:** 1 Max Planck Institute for Polymer Research, Ackermannweg 10, 55128 Mainz, Germany; 2 Physics Department, University of Trento, via Sommarive 14, I-38123 Trento, Italy; 3 INFN-TIFPA, Trento Institute for Fundamental Physics and Applications, I-38123 Trento, Italy

**Keywords:** computer simulations, finite-size effects, calculation of free energies, thermodynamic limit

## Abstract

The spatial block analysis (SBA) method has been introduced to efficiently extrapolate thermodynamic quantities from finite-size computer simulations of a large variety of physical systems. In the particular case of simple liquids and liquid mixtures, by subdividing the simulation box into blocks of increasing size and calculating volume-dependent fluctuations of the number of particles, it is possible to extrapolate the bulk isothermal compressibility and Kirkwood–Buff integrals in the thermodynamic limit. Only by explicitly including finite-size effects, ubiquitous in computer simulations, into the SBA method, the extrapolation to the thermodynamic limit can be achieved. In this review, we discuss two of these finite-size effects in the context of the SBA method due to (i) the statistical ensemble and (ii) the finite integration domains used in computer simulations. To illustrate the method, we consider prototypical liquids and liquid mixtures described by truncated and shifted Lennard–Jones (TSLJ) potentials. Furthermore, we show some of the most recent developments of the SBA method, in particular its use to calculate chemical potentials of liquids in a wide range of density/concentration conditions.

## 1. Introduction

In the last few decades, computational studies of soft matter have gained ground in the no-man’s land between purely theoretical studies and experimental investigations. Arguably, this success is due to the use of statistical mechanics relations between macroscopic thermodynamic properties and microscopic components and interactions of a physical system in the thermodynamic limit (TL) [1,2]. However, and apart from a few examples [3,4,5], computer simulations are mainly constrained to consider closed systems with a finite and usually small number of particles $N_{0}$. These limitations introduce spurious finite-size effects, apparent in the simulation results, that in spite of the current computing capabilities are still the subject of intense investigations [6,7,8,9,10,11,12,13].

A meaningful comparison between computer simulations of finite systems and experimental results has been always a difficult task. In principle, it is possible to extrapolate the simulation data to the quantities of interest in the thermodynamic limit by considering systems of increasing size and performing simulations for each of them. The SBA method has been proposed as a more efficient alternative where only one system is examined and then subdivided into blocks of different size from which the data are extracted. The method is rather general since it was originally proposed to study the critical behavior of Ising systems [14,15] and then extended to study liquids [16,17,18,19,20,21] and even the elastic constants of model solids [22].

In this paper, we examine the SBA method focusing on the extrapolation of bulk thermodynamic properties of simple liquids. We use prototypical liquids and mixtures described by truncated and shifted Lennard–Jones (TSLJ) potentials to discuss the original ideas [16,17] and explore the background [20,21,23,24,25,26] for the most recent developments [6,7,9] of the method. The simple examples presented here, in addition to the results available in the literature [6], suggest that the method is suitable for the calculation of trends in the chemical potential of complex liquids in a wide range of density/concentration conditions.

The paper is organized as follows: In Section 2, we introduce the relevant finite-size effects present in standard computer simulations. In Section 3, we introduce the finite-size integral equations for liquids and illustrate the procedure to extrapolate thermodynamic quantities. In Section 4, we discuss the extension of the block analysis method to liquid mixtures. We conclude the paper in Section 5.

## 2. Boundary and Ensemble Finite-Size Effects

Statistical mechanics establishes the connection between macroscopic thermodynamic properties and the microscopic components and interactions of a physical system. An interesting example of this relation is provided by the compressibility equation that identifies the density fluctuations of a system in the grand canonical ensemble with the bulk isothermal compressibility $\kappa_{T}$ [27]. In the thermodynamic limit (TL), the isothermal compressibility of a homogeneous system is related to the fluctuations of the number of particles via the expression [1]:

(1) $$\chi_{T}^{\infty }=\frac{\langle N^{2}\rangle -{\langle N\rangle }^{2}}{\langle N\rangle }\;,$$

with 〈N〉 the average number of particles contained in a volume *V* of the fluid. The reduced isothermal compressibility $\chi_{T}^{\infty }=\rho k_{B}T\kappa_{T}$ is the ratio between the bulk isothermal compressibility of the system, $\kappa_{T}$, and the isothermal compressibility of the ideal gas ${\left(\rho k_{B}T\right)}^{-1}$ with ρ=〈N〉/V.

**Figure 1:**
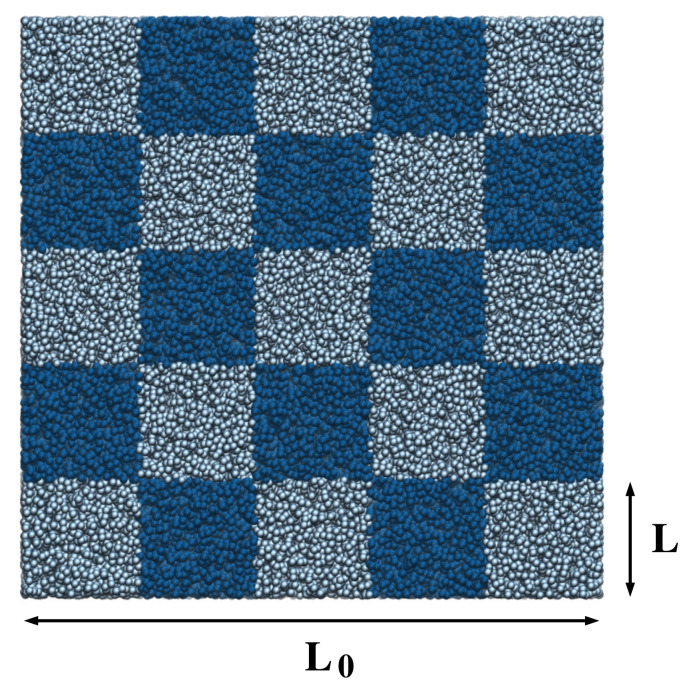
Snapshot of the simulation box for a system of particles interacting via a TSLJ potential at density $\rho \sigma^{3}=0.1$ and temperature $k_{B}T=1.2\epsilon$. In this particular example, a box of linear size $L_{0}$ has been subdivided into blocks of linear dimension $L=L_{0}/5$ as indicated by the different color shades. The figure has been rendered with the Visual Molecular Dynamics (VMD) program [31].

Various finite-size effects can be included in the block analysis aiming at extrapolating interesting thermodynamic quantities. In practice, let us consider a system of $N_{0}$ particles where the simulation box of volume $V_{0}=L_{0}^{3}$ is divided into subdomains of volume $V=L^{3}$, as illustrated in Figure 1. By evaluating the fluctuations of the number of particles in these subdomains, it is possible to obtain the distribution $P_{L,L_{0}}\left(N\right)$ of the number of particles, with *k*-moments given by [25]:

(2) $${\langle N^{k}\rangle }_{L,L_{0}}=\sum_{N=0}^{N_{0}}N^{k}\;P_{L,L_{0}}\left(N\right)\;.$$

The second moment of the distribution is related to the reduced isothermal compressibility of the finite system $\chi_{T}\left(L,L_{0}\right)$ [14,16,17,25]:

(3) $$\chi_{T}\left(L,L_{0}\right)=\frac{{\langle N^{2}\rangle }_{L,L_{0}}-{\langle N\rangle }_{L,L_{0}}^{2}}{{\langle N\rangle }_{L,L_{0}}}\;.$$

The finite-size reduced isothermal compressibility, $\chi_{T}\left(L,L_{0}\right)$, can be extrapolated to the reduced isothermal compressibility in the TL, $\chi_{T}^{\infty }$, taking the limits $L,L_{0}\to \infty$. Originally [17,25], by applying periodic boundary conditions (PBCs) to the total linear size $L_{0}$ and taking into account volumes such that L≫ζ with ζ the correlation length of the system, it has been proposed that the difference between $\chi_{T}\left(L,L_{0}\right)$ and $\chi_{T}^{\infty }$ is related to boundary effects associated with the finite-size of the subdomains. This difference takes the form [16,17]:

(4) $$\chi_{T}\left(L,L_{0}\to \infty \right)=\chi_{T}^{\infty }+\frac{c}{L}+O\left(\frac{1}{L^{2}}\right)\;,$$

with *c* a constant. Recently, Equation (4) has been obtained [28] using arguments based on the thermodynamics of small systems [29,30], underpinning the consistency of the result.

To investigate this expression, we consider a liquid system whose potential energy is described by a 12–6Lennard–Jones potential truncated, with cutoff radius $r_{c}/\sigma =2^{1/6}$, and shifted. The parameters ϵ, σ and *m*, define the units of energy, length and mass, respectively. All the results are expressed in LJ units with time $\sigma {\left(m/\epsilon \right)}^{1/2}$, temperature $\epsilon /k_{B}$ and pressure $\epsilon /\sigma^{3}$. Various system sizes, namely $N_{0}=10^{4},10^{5}$ and $10^{6}$, are considered, and the density is fixed at $\rho \sigma^{3}=0.864$, thus defining the linear size of the simulation box $L_{0}$. The systems are equilibrated at $k_{B}T=1.2\epsilon$, enforced with a Langevin thermostat with damping coefficient $\gamma (\sigma {\left(m/\epsilon \right)}^{1/2})=1.0$, for $2\times 10^{6}$ molecular dynamics (MD) steps using a time step of $\delta t/\left(\sigma {\left(m/\epsilon \right)}^{1/2}\right)=10^{-3}$. Production runs span $10^{6}$ MD steps. All the simulations have been performed with the ESPResSo++ [32] simulation package.

To use the block analysis method, we compute the fluctuations of the number of particles. In particular, we choose domains of size $1<L/\sigma <L_{0}/\sigma$ to scan continuously the fluctuations as a function of domain size. To increase the amount of statistics, we use 100 randomly-positioned subdomains per simulation frame.

**Figure 2:**
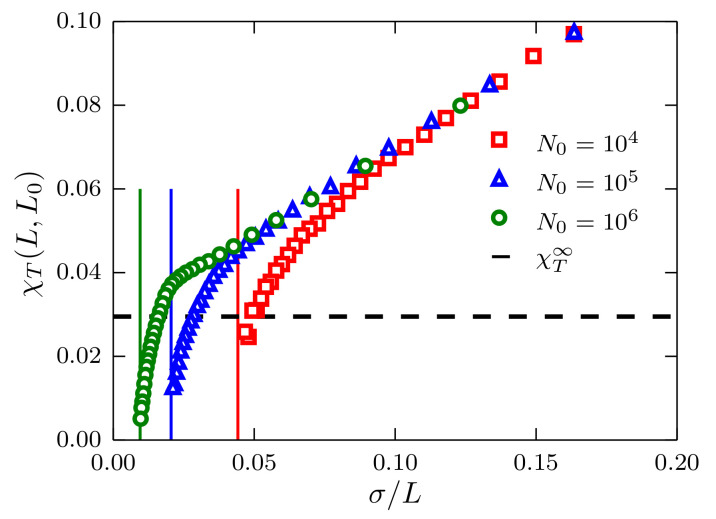
Fluctuations of the number of particles $\chi_{T}\left(L,L_{0}\right)$ as a function of σ/L for systems described by a TSLJ potential with $r_{c}/\sigma =2^{1/6}$. Data corresponding to system sizes $N_{0}=10^{4},10^{5}$ and $10^{6}$ are presented using red squares, blue triangles and green circles, respectively. The vertical lines indicate the limit $\sigma /L_{0}$ at which fluctuations become zero. The black horizontal dashed line indicates the value $\chi_{T}^{\infty }=\rho k_{B}T\kappa_{T}=0.0295$ with $\kappa_{T}$ the bulk compressibility obtained with the method described in [6].

In Figure 2, we report $\chi_{T}\left(L,L_{0}\right)$ as a function of σ/L. The linear behavior predicted in Equation (4) is apparent for $L\ll L_{0}$. There are evident deviations from the linear behavior, which are not included in Equation (4), since this equation has been obtained for a system in the grand canonical ensemble. As a matter of fact, the deviations from linearity are mainly related to the fixed size of the system because when $L\to L_{0}$, $\chi_{T}\left(L_{0},L_{0}\right)=0$, that is, the fluctuations of the number of particles for a closed system are equal to zero. In principle, the isothermal compressibility in the TL can be extracted by extrapolating a line to the *y*-axis, i.e., σ/L→0, and determining the *y*-intercept. This procedure, however, might lead to ambiguous and strongly-size-dependent results as suggested by the same plot.

From the previous discussion, Equation (4) satisfactorily describes the boundary size effects present in a system described in the grand canonical ensemble. However, ensemble size effects, i.e., the fact that we are computing quantities defined in the grand canonical ensemble using information obtained from a system in a canonical ensemble, are important even in cases where the size of the system might appear to be enormous ($L_{0}/\sigma =105$ for $N_{0}=10^{6}$ where ζ/σ≈10).

It is thus clear that the isothermal compressibility of a finite-size system in the TL, i.e., $L,L_{0}\to \infty$ with $\rho =N_{0}/L_{0}^{3}$, should equate to the bulk isothermal compressibility $\kappa_{T}$. An elegant analysis using probabilistic arguments for the ideal gas case [26,33] shows that the finite-size reduced isothermal compressibility can be written as:

(5) $$\chi_{T}\left(L,L_{0}\right)=\chi_{T}^{\infty }\left(1-{\left(\frac{L}{L_{0}}\right)}^{3}\right)\;.$$

**Figure 3:**
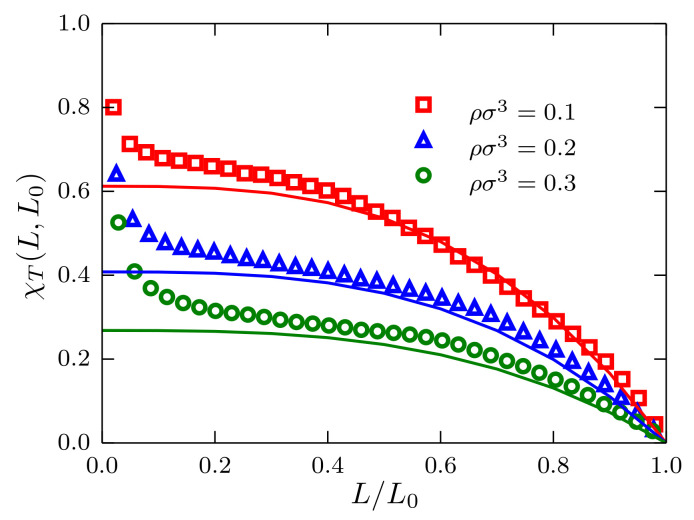
Fluctuations of the number of particles $\chi_{T}\left(L,L_{0}\right)$ as a function of the ratio $L/L_{0}$ for systems described by a TSLJ potential with $r_{c}/\sigma =2^{1/6}$. Results corresponding to systems of $N_{0}=10^{5}$ particles with densities $\rho \sigma^{3}=0.1,\;0.2$ and 0.3 are presented using red squares, blue triangles and green circles, respectively. The theoretical prediction presented in the text is plotted using the corresponding value for $\chi_{T}^{\infty }$, obtained as described in [6], and solid-line curves with the same color code.

In spite of the simplicity of the system chosen in this study, it cannot be identified with the ideal gas. However, at very low densities and temperature $k_{B}T=1.2\epsilon$, the system behaves more like a real gas, and a meaningful trend could be identified. Therefore, to investigate Equation (5), we consider the density range $\rho \sigma^{3}=0.1\;,\;\cdots \;,\;1.0$ for systems of size $N_{0}=10^{5}$ particles. Results are presented in Figure 3 for the cases $\rho \sigma^{3}=0.1,\;0.2$ and 0.3. The three datasets follow the theoretical prediction in Equation (5) with deviations from this behavior for $L\ll L_{0}$, thus indicating the signature of boundary finite-size effects. As expected, the data presented also suggest that upon increasing density, the deviations from the ideal gas behavior become more evident, as can be seen in the case $\rho \sigma^{3}=0.3$.

**Figure 4:**
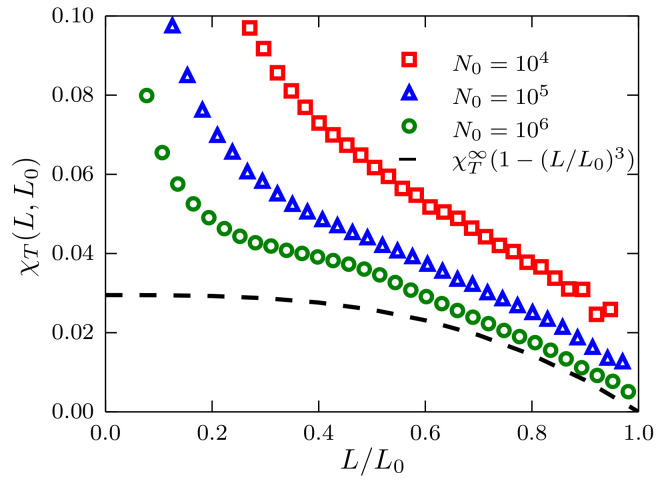
Fluctuations of the number of particles $\chi_{T}\left(L,L_{0}\right)$ as a function of the ratio $L/L_{0}$ for systems described by a TSLJ potential with $r_{c}/\sigma =2^{1/6}$. Results corresponding to sizes $N_{0}=10^{4},\;10^{5}$ and $10^{6}$, with density $\rho \sigma^{3}=0.864$, using red squares, blue triangles and green circles, respectively. The theoretical prediction presented in the text is plotted as the black dashed curve using $\chi_{T}^{\infty }=0.0295$.

This is also seen in Figure 4, where for a system with density $\rho \sigma^{3}=0.864$, the deviations from the ideal gas case are much more evident. As a matter of fact, even for the largest size considered ($N_{0}$ = $10^{6}$), it is not possible to convincingly reproduce the ideal gas behavior.

Nonetheless, one intuitively could imagine that the following expression:

(6) $$\chi_{T}\left(L,L_{0}\right)=\chi_{T}^{\infty }\left(1-{\left(\frac{L}{L_{0}}\right)}^{3}\right)+\frac{c}{L}+O\left(\frac{1}{L^{2}}\right)\;,$$

captures the two finite-size effects, ensemble and boundary [25]. By neglecting the $O(1/L^{2})$ terms, defining $\lambda =L/L_{0}$ and multiplying everything by λ, we obtain:

(7) $$\lambda \chi_{T}\left(\lambda \right)=\lambda \chi_{T}^{\infty }\left(1-\lambda^{3}\right)+\frac{c}{L_{0}}\;.$$

**Figure 5:**
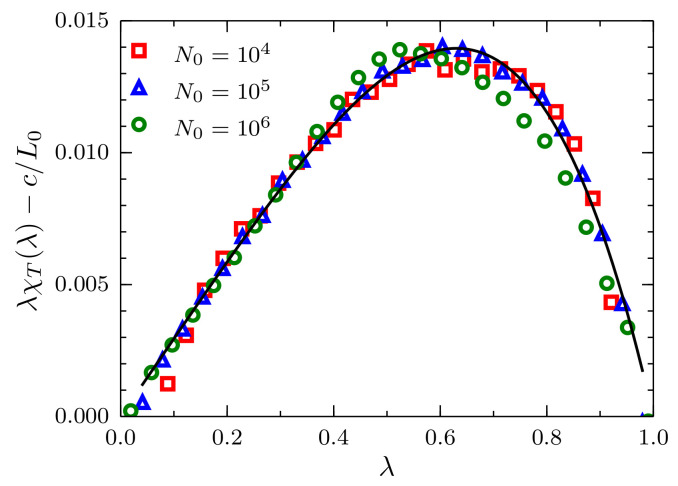
Scaled fluctuations of the number of particles $\lambda \chi_{T}\left(L,L_{0}\right)$, minus $c/L_{0}$, versus the ratio $\lambda =L/L_{0}$ for systems described by a TSLJ potential with $r_{c}/\sigma =2^{1/6}$. Results corresponding to sizes $N_{0}=10^{4},\;10^{5}$ and $10^{6}$, with density $\rho \sigma^{3}=0.864$, using red squares, blue triangles and green circles, respectively. The theoretical prediction Equation (7) presented in the text is plotted as the black solid curve using $\chi_{T}^{\infty }=0.0295$ and c=0.415σ.

Equation (7) is more convenient to analyze because in the limit λ→0, provided that $\zeta <L<L_{0}$, $\lambda^{3}$ is negligible, and this expression can be approximated to a linear function in λ with slope $\chi_{T}^{\infty }$ and *y*-intercept equal to $c/L_{0}$. In particular, we use a simple linear regression in the interval 0.0<λ<0.3, with the fluctuations data for $N_{0}=10^{5}$, to find $\chi_{T}^{\infty }=0.0295\left(5\right)$ and c=0.415(5)σ. Results of the scaled fluctuations $\lambda \chi_{T}\left(\lambda \right)$ minus $c/L_{0}$ are presented in Figure 5, where the intensive character of the constant *c* becomes clear. By replacing the calculated values $\chi_{T}^{\infty }$ and *c* in Equation (7), we obtain the black curve that superimposes on the simulation data in the full range 0<λ<1.

In addition to the explicit finite-size effects discussed above, there is another type of effect related to the periodicity of the simulation box. This is the case of implicit finite-size effects that appear due to anisotropies in the pair correlation function of the system, generated by the use of PBCs [34,35]. These effects, extremely important for small simulation setups, appear as oscillations in $\lambda \chi_{T}\left(\lambda \right)$ for λ≈1 caused by short range interactions between the system and its nearest neighbor images. However, given the large sizes of the systems considered here, implicit finite-size effects can be safely ignored in the present discussion.

**Figure 6:**
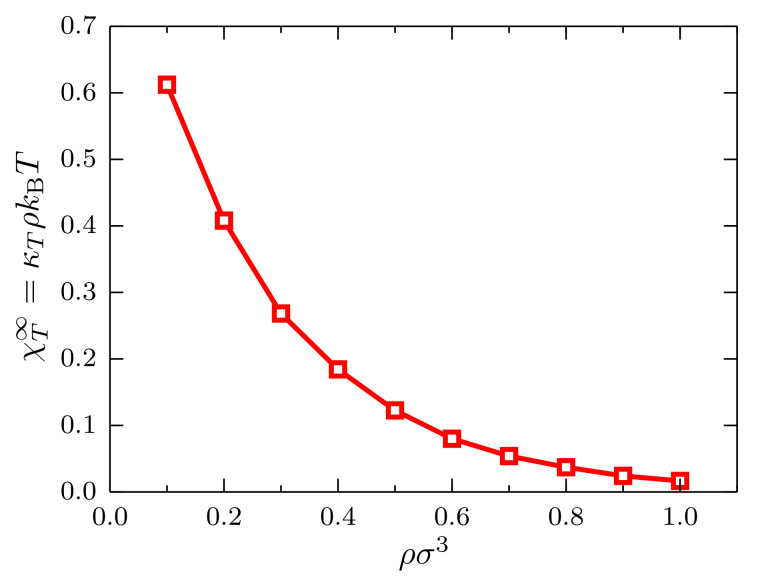
Ratio $\chi_{T}^{\infty }=\kappa_{T}/\kappa_{T}^{IG}$ at $k_{B}T=1.2\epsilon$ as a function of the density for systems described by a TSLJ potential with $r_{c}/\sigma =2^{1/6}$, with $\kappa_{T}^{IG}={\left(\rho k_{B}T\right)}^{-1}$ the isothermal compressibility of the ideal gas. The red curve is a guide to the eye.

With the trajectories of the system with $N_{0}=10^{5}$ particles in the density interval $0.1<\rho \sigma^{3}\;<\;1.0$, we compute the scaled fluctuations $\lambda \chi_{T}\left(\lambda \right)$ and determine, as before, the ratio $\chi_{T}^{\infty }=\kappa_{T}/\kappa_{T}^{IG}$ as a function of the density, with $\kappa_{T}^{IG}={\left(\rho k_{B}T\right)}^{-1}$ the isothermal compressibility of the ideal gas (see Figure 6). As expected for this system at $k_{B}T=1.2\epsilon$, a monotonically-decreasing behavior is observed since the system becomes less compressible as the density increases.

The isothermal compressibility as a function of the density allows one to investigate more interesting thermodynamic properties, as has been recently demonstrated [6,7]. For example, the isothermal compressibility can be written as:

(8) $$\kappa_{T}=\frac{1}{\rho^{2}}{\left.\frac{\partial \rho }{\partial \mu }\right|}_{T}\;,$$

which can be rearranged, in terms of the chemical potential μ, as:

(9) $$\delta \mu =\int_{\rho_{0}}^{\rho }\frac{d\rho^{\prime }}{\rho^{\prime 2}\kappa_{T}}\;$$

with $\delta \mu =\mu -\mu_{0}$ and $\mu_{0}$ the chemical potential of the system at the reference density $\rho_{0}$. In practice, one usually is interested in the excess chemical potential (In this context, the word excess should be replaced with residual. The residual chemical potential is the difference between the chemical potential of the target system and that of an ideal gas at the same density, temperature and composition. We misuse the expression excess chemical potential to match the modern literature.):

(10) $$\delta \mu^{\mathrm{ex}}=\delta \mu -k_{B}T\ln\rho ,$$

obtained by subtracting from δμ the density-dependent part of the chemical potential of the ideal gas.

To validate the results obtained using Equation (10), it is necessary to use a different computational method to evaluate $\mu_{0}$. For that purpose, any computational method aiming at calculating chemical potentials could be used. In particular, we use the spatially-resolved thermodynamic integration (SPARTIAN) method [36], recently implemented by us. In SPARTIAN, the target system, described with atomistic resolution, is embedded in a reservoir of ideal gas particles. An interface between the two subdomains is defined such that molecules are free to diffuse, adapting their resolution on the fly. A uniform density across the simulation box is guaranteed by applying a single-molecule external potential that is identified with the difference in chemical potential between the two resolutions, i.e., the excess chemical potential of the target system. This method has been validated by calculating excess chemical potentials for Lennard–Jones liquids, mixtures, as well as for simple point-charge (SPC) and extended simple point-charge (SPC/E) water models and aqueous sodium chloride solutions, all in good agreement with state-of-the-art computational methods.

**Figure 7:**
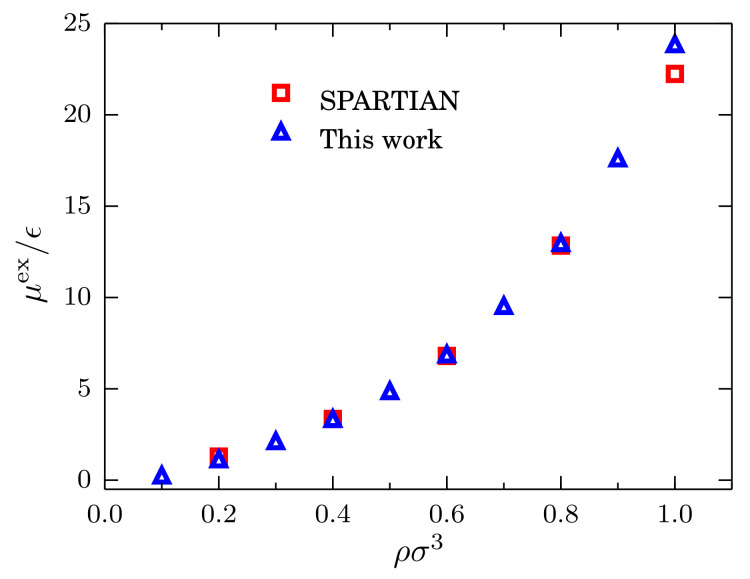
Excess chemical potential $\mu^{ex}/\epsilon$ at $k_{B}T=1.2\epsilon$ as a function of the density for systems described by a TSLJ potential with $r_{c}/\sigma =2^{1/6}$. Red squares indicate the data obtained with the spatially-resolved thermodynamic integration (SPARTIAN) method [36], and the blue triangles are the data points obtained with the method outlined in the text.

For the comparison, we consider the same system at the same temperature with densities $\rho \sigma^{3}$ = 0.2, 0.4, 0.6, 0.8 and 1.0. Results for the excess chemical potential as a function of the density are presented in Figure 7 where the value of $\rho_{0}\sigma =0.6$ has been used as the reference value. Once $\delta \mu^{ex}$ is rescaled, it becomes clear that the agreement between the two methods is remarkable. This result suggests that the simple calculation of the fluctuations of the number of particles, used in combination with Equation (7), provides us with an efficient and accurate method to compute the chemical potential of simple liquids, which can be extended to more complex fluids [6].

In this section, Equation (7) has been introduced in a rather intuitive manner. However, the presented results suggest that it encompasses the relevant finite-size effects of the system and allows one to compute bulk thermodynamic quantities. In the following section, we derive Equation (7) more rigorously and explore, using a different example, its range of validity.

## 3. Finite-Size Ornstein–Zernike Integral Equation

Fluctuations of the number of particles are related to the local structure of a liquid. Let us consider a molecular liquid of average density ρ at temperature *T* in equilibrium with a reservoir of particles, i.e., an open system. The fluctuations of the number of molecules are related to the local structure of the liquid via the Ornstein–Zernike integral equation [1,37]:

(11) $$\frac{\Delta^{2}\left(N\right)}{\langle N\rangle }=1+\frac{\rho }{V}\int_{V}\int_{V}\left[g^{o}\left(r_{1},r_{2}\right)-1\right]\;dr_{1}\;dr_{2}\;,$$

where $\Delta^{2}\left(N\right)/\langle N\rangle$ are the fluctuations of the number of particles, $\Delta^{2}\left(N\right)=\langle N^{2}\rangle -{\langle N\rangle }^{2}$ and $g^{o}\left(r_{1},r_{2}\right)$ is the pair correlation function of the open system and $r_{1},r_{2}$ the position vectors of a pair of fluid particles. To solve the integral in Equation (11), one assumes that the fluid is homogeneous, isotropic and that the system is in the thermodynamic limit (TL), i.e., V→∞, 〈N〉→∞ with ρ=〈N〉/V= constant. An infinite, homogeneous and isotropic system is translationally invariant; therefore, we rewrite Equation (11) as [1]:

(12) $$\chi_{T}^{\infty }=\frac{\Delta^{2}\left(N\right)}{\langle N\rangle }=1+4\pi \rho \int_{0}^{\infty }\left(g^{o}\left(r\right)-1\right)\;r^{2}\;dr\;,$$

with $\chi_{T}^{\infty }=\rho k_{B}T\kappa_{T}$, $\kappa_{T}$ being the isothermal compressibility of the bulk system. We have replaced $g^{o}\left(r_{1},r_{2}\right)$ with $g^{o}\left(r\right)$ the radial distribution function (RDF) of the open system, with $r=|r_{2}-r_{1}|$.

An alternative version of the OZ integral equation for finite systems has been introduced [25]. For a finite system with total volume $V_{0}$ with PBCs we have:

(13) $$\chi_{T}\left(V,V_{0}\right)=\frac{\Delta^{2}\left(N;V,V_{0}\right)}{{\langle N\rangle }_{V,V_{0}}}=1+\frac{\rho }{V}\int_{V}\int_{V}\left[g^{c}\left(r_{12}\right)-1\right]\;dr_{1}\;dr_{2}\;,$$

where $g^{c}\left(r_{12}\right)$, $r_{12}=\left|r_{2}-r_{1}\right|$, is the pair correlation function of the closed system with total number of particles $N_{0}$, and $\Delta^{2}\left(N;V,V_{0}\right)={\langle N^{2}\rangle }_{V,V_{0}}-{\langle N\rangle }_{V,V_{0}}^{2}$. The fluctuations of the number of particles thus depend on both subdomain and simulation box volumes.

For a single component fluid of density ρ at temperature *T* with fixed number of particles $N_{0}$ and volume $V_{0}$, its RDF can be written in terms of an expansion around $N_{0}$ as [23,24,25,26,33]:

(14) $$\begin{array}{cc} g^{c}\left(r\right)= & g^{o}\left(r\right)-\frac{\chi_{T}^{\infty }}{N_{0}}\;. \end{array}$$

As a matter of fact, the expansion includes terms that depend on the partial derivative of $g^{o}\left(r\right)$ with respect to the density. However, we anticipate here that for the present analysis, their contribution is negligible [6]. By replacing $g^{c}\left(r\right)$ in the integral on the r.h.s of Equation (13), we obtain:

(15) $$\frac{\rho }{V}\int_{V}\int_{V}\left(g^{c}\left(r_{12}\right)-1\right)\;dr_{1}\;dr_{2}=I_{V,V}-\frac{V}{V_{0}}\chi_{T}^{\infty }\;,$$

where:

(16) $$I_{V,V}=\frac{\rho }{V}\int_{V}\int_{V}\left(g^{o}\left(r_{12}\right)-1\right)\;dr_{1}\;dr_{2}\;,$$

and we use that $\rho =N_{0}/V_{0}$.

Next, we include explicitly the second finite-size effect, i.e., the fact that the volume *V* is finite and embedded into a finite volume $V_{0}$ with PBCs. For this, we rewrite $I_{V,V}$ as [17]:

$$I_{V,V_{0}-V}=I_{V,V_{0}}-I_{V,V}\;,$$

with:

$$\begin{array}{cc} I_{V,V_{0}} & =\frac{\rho }{V}\int_{V}\int_{V_{0}}\left(g^{o}\left(r_{12}\right)-1\right)\;dr_{1}\;dr_{2}\;\\ I_{V,V_{0}-V} & =\frac{\rho }{V}\int_{V}\int_{V_{0}-V}\left(g^{o}\left(r_{12}\right)-1\right)\;dr_{1}\;dr_{2}\;. \end{array}$$

As pointed out by Rovere, Heermann and Binder [17], the two integrals $I_{V,V}$ and $I_{V,V_{0}}$ are equal when $r_{1}$ and $r_{2}$ are both within the volume *V*. When $r_{12}>\zeta$, the integrand $(g^{o}\left(r_{12}\right)-1)=0$, and it does not contribute to the integrals. Close to the boundary of the subdomain *V*, for $r_{12}<\zeta$, and in particular when $r_{1}$ lies inside and $r_{2}$ outside the volume *V*, there are contributions missing in $I_{V,V}$, which are present in $I_{V,V_{0}}$. Therefore, the difference between the two integrals $I_{V,V_{0}-V}=I_{V,V_{0}}-I_{V,V}$ must be proportional to the surface volume ratio of the subdomain *V* [17], i.e.,

(17) $$I_{V,V_{0}-V}=\frac{c_{1}}{L}+{\left(\frac{c_{2}}{L}\right)}^{2}+O\left(\frac{1}{L^{3}}\right)\;,$$

with $c_{1}$, $c_{2}$ proportionality constants with units of length that, at this point, we assume to be intensive.

To compute $I_{V,V_{0}}$, we require that $\zeta <L<L_{0}$. Since we assume PBCs, the system is translationally invariant. Hence, upon applying the transformation $r_{12}\to r=r_{2}-r_{1}$, the expression:

(18) $$I_{V,V_{0}}=\rho \int_{V_{0}}\left(g^{o}\left(r\right)-1\right)\;dr=\chi_{T}^{\infty }-1\;$$

is obtained, where we assume that $g^{o}\left(r>\zeta \right)=1$, thus ignoring fluctuations of the RDF beyond the volume *V*. By combining these two results, we obtain:

(19) $$I_{V,V}=\chi_{T}^{\infty }-1+\frac{c_{1}}{L}+{\left(\frac{c_{2}}{L}\right)}^{2}\;,$$

and by including this result in Equation (15), we arrive at the following expression:

(20) $$\frac{\rho }{V}\int_{V}\int_{V}\left(g^{c}\left(r_{12}\right)-1\right)\;dr_{1}\;dr_{2}=\chi_{T}^{\infty }\left(1-{\left(\frac{L}{L_{0}}\right)}^{3}\right)-1+\frac{c_{1}}{L}+{\left(\frac{c_{2}}{L}\right)}^{2}\;.$$

Finally, this expression becomes:

(21) $$\chi_{T}\left(L,L_{0}\right)=\chi_{T}^{\infty }\left(1-{\left(\frac{L}{L_{0}}\right)}^{3}\right)+\frac{c_{1}}{L}+{\left(\frac{c_{2}}{L}\right)}^{2}\;,$$

and by defining $\lambda =L/L_{0}$, we write:

(22) $$\lambda \chi_{T}\left(\lambda \right)=\lambda \chi_{T}^{\infty }\left(1-\lambda^{3}\right)+\frac{c_{1}}{L_{0}}+{\left(\frac{c_{2}}{L_{0}}\right)}^{2}\frac{1}{\lambda }\;.$$

Equations (7) and (22) differ in the $c_{2}^{2}/L_{0}^{2}\lambda$ term that appears from considering the boundary finite-size effects. One possible scenario in which this difference might play a role is in the case of simulations near critical conditions where the correlation length of the system tends to infinity.

To test this expression, we perform simulations of systems with potential energy described by the truncated, at $r_{c}/\sigma =2.5$, and shifted 12–6 Lennard–Jones potential. We consider systems with $N_{0}=24,000$ particles, with densities spanning the range $0.05<\rho \sigma^{3}<0.70$. Two temperatures were considered, $k_{B}T=2.00\epsilon$ and 1.15ϵ. The critical point of this system has been reported at $\rho_{c}\sigma^{3}=0.319$ and $k_{B}T_{c}=1.086\epsilon$ [38].

**Figure 8:**
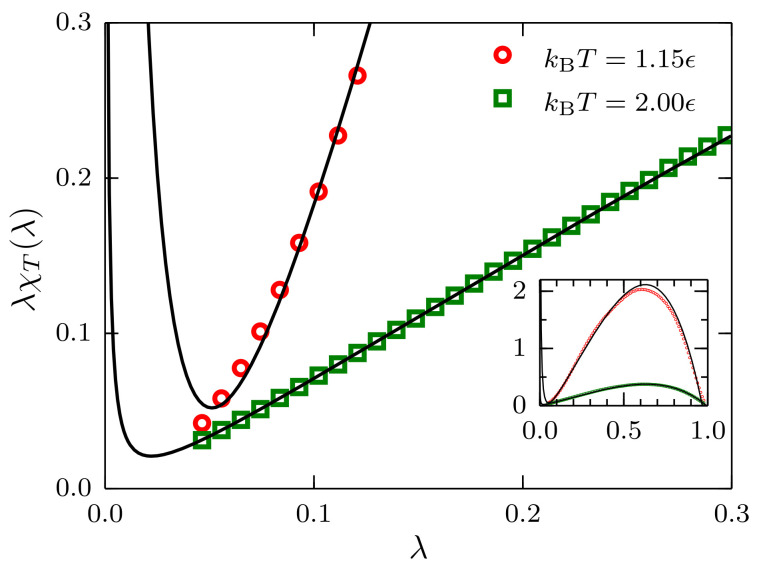
Reduced fluctuations as a function of λ for systems described by a TSLJ potential with $r_{c}/\sigma =2.5$ with density $\rho \sigma^{3}=0.3$ at temperatures $k_{B}T=2.00\epsilon$ and 1.15ϵ. For the latter case, it is apparent that the contribution proportional to $\lambda^{-1}$ is not negligible. The inset shows the full range 0<λ<1. The black curves are the result of fitting the data to Equation (22).

We report the reduced fluctuations $\lambda \chi_{T}\left(\lambda \right)$ as a function of λ for $\rho \sigma^{3}=0.3$ in Figure 8. In the case $k_{B}T=2.00\epsilon$, the effect of the $\lambda^{-1}$ term in Equation (22) is negligible, and a linear approximation in the region λ<0.3 seems to be well justified. However, for the case close to the critical point, i.e., $k_{B}T=1.15\epsilon$, the effect of this term is evident and should be included in the extrapolation to $\chi_{T}^{\infty }$.

**Figure 9:**
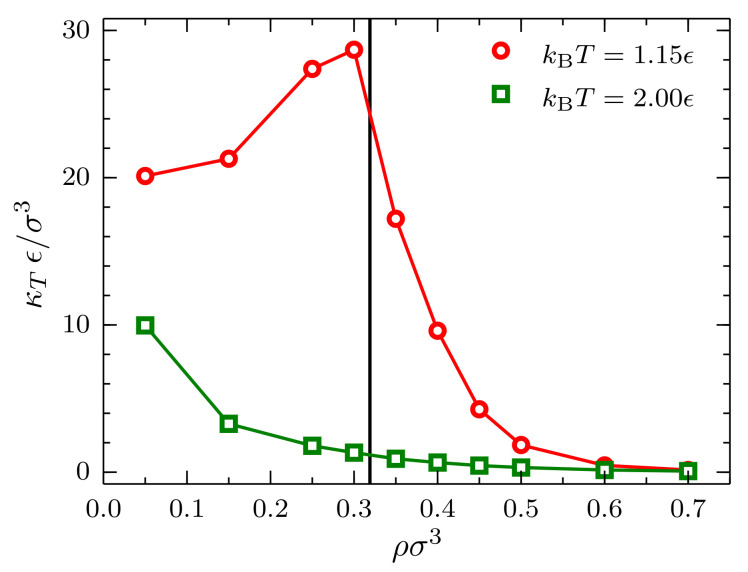
Bulk isothermal compressibility $\kappa_{T}$ as a function of the density ρ at $k_{B}T=1.15\epsilon$ (red circles) and $k_{B}T=2.00\epsilon$ (green squares) for systems described by a TSLJ potential with $r_{c}/\sigma =2.5$. The vertical black line indicates the location of the critical density $\rho \sigma^{3}=0.319$ [38].

Finally, upon extrapolating to $\chi_{T}^{\infty }$, an interesting behavior is observed for the bulk isothermal compressibility $\kappa_{T}$ as a function of density (Figure 9). In the case $k_{B}T=2.00\epsilon$, as expected, a monotonically-decreasing behavior with increasing density is observed. More interestingly, in the case $k_{B}T=1.15\epsilon$, the monotonically-decreasing behavior is interrupted by a singularity in the isothermal compressibility in the vicinity of the critical density. This cusp in the curve is expected since the isothermal compressibility of a fluid at the critical point is infinite.

The use of finite-size integral equations is general enough to admit generalizations of other systems of interest. In the next section, we describe one of such possible extensions: the study of binary mixtures.

## 4. Mixtures

Kirkwood–Buff (KB) theory [39] is arguably the most successful framework to investigate the properties of liquid mixtures that relates the local structure of a system to density fluctuations in the grand canonical ensemble. These quantities are in turn related to equilibrium thermodynamic quantities such as the compressibility, the partial molar volumes and the derivatives of the chemical potentials [2]. Formulated more than sixty years ago, KB enjoys renewed interest in the computational soft-matter and statistical physics communities [6,7,9,10,11,12,13]. Recent works have shown promising applications related to solvation of biomolecules [40] and potential uses to compute multicomponent diffusion in liquids [41] and to study complex phenomena such as self-assembly of proteins [42] and polymer conformation in complex mixtures [4,43].

For a multicomponent fluid of species i,j in equilibrium at temperature *T*, the Kirkwood–Buff integral (KBI) is defined as:

(23) $$G_{ij}^{o}=V\left(\frac{\langle N_{i}N_{j}\rangle -\langle N_{i}\rangle \langle N_{j}\rangle }{\langle N_{i}\rangle \langle N_{j}\rangle }-\frac{\delta_{ij}}{\langle N_{i}\rangle }\right)=\frac{1}{V}\int_{V}\int_{V}\left[g_{ij}^{o}\left(r_{12}\right)-1\right]\;dr_{1}\;dr_{2}\;,$$

with $\delta_{ij}$ the Kronecker delta. The superscript (o) indicates that this definition holds for an open system, i.e., a system in the grand canonical ensemble. In practice, we compute fluctuations of the number of particles in a subdomain of volume *V* embedded in a reservoir whose size goes to infinity. Thus, $\langle N_{i}\rangle$ is the average number of *i*-particles inside *V*, or $\rho_{i}=\langle N_{i}\rangle /V$. $g_{ij}^{o}\left(r_{12}\right)$ is the multicomponent radial distribution function (RDF) of the infinite system, with $r_{12}=r_{2}-r_{1}$.

Let us recall that in computer simulations one considers systems with total fixed number of particles $N_{0}$ and volume $V_{0}$ with PBCs. In this case, we have [35]:

(24) $$G_{ij}\left(L,L_{0}\right)=V\left(\frac{{\langle N_{i}N_{j}\rangle }^{\prime }-{\langle N_{i}\rangle }^{\prime }{\langle N_{j}\rangle }^{\prime }}{{\langle N_{i}\rangle }^{\prime }{\langle N_{j}\rangle }^{\prime }}-\frac{\delta_{ij}}{{\langle N_{i}\rangle }^{\prime }}\right)=\frac{1}{V}\int_{V}\int_{V}\left[g_{ij}^{c}\left(r_{12}\right)-1\right]\;dr_{1}\;dr_{2}\;.$$

The finite-size KBI $G_{ij}\left(L,L_{0}\right)$ is evaluated by computing fluctuations of the number of particles in finite subdomains of volume *V* inside a simulation box of volume $V_{0}$. The average number of *i*-particles ${\langle N_{i}\rangle }^{\prime }\equiv {\langle N_{i}\rangle }_{V,V_{0}}$ depends on both subdomain and simulation box volumes. Moreover, the integral on the r.h.s. of Equation (24) should be evaluated for the RDF of the finite system $g_{ij}^{c}\left(r_{12}\right)$ with volume $V_{0}$ by using a finite integration domain *V*.

As has been done for the single component case, we include in this example both, ensemble and boundary, finite-size effects. For the former, the following correction has been suggested [44]:

(25) $$g_{ij}^{c}\left(r\right)=g_{ij}^{o}\left(r\right)-\frac{1}{V_{0}}\left(\frac{\delta_{ij}}{\rho_{i}}+G_{ij}^{\infty }\right)\;,$$

based on the asymptotic limit $g_{ij}^{c}\left(r\gg \zeta \right)=1-\left(\delta_{ij}/\rho_{i}+G_{ij}^{\infty }\right)/V_{0}$ discussed in [2]. As expected, when the total volume $V_{0}\to \infty$, we recover $g_{ij}^{c}\left(r\right)=g_{ij}^{o}\left(r\right)$. By including Equation (25) in the integral on the r.h.s. of Equation (24) and evaluating the finite-size integral as for the single component case, we finally obtain:

(26) $$\lambda G_{ij}\left(\lambda \right)=\lambda G_{ij}^{\infty }\left(1-\lambda^{3}\right)-\lambda^{4}\frac{\delta_{ij}}{\rho_{i}}+\frac{\alpha_{ij}}{L_{0}}\;,$$

with $\lambda \equiv L/L_{0}$ and $\alpha_{ij}$ an intensive parameter with units of length. In the limit $L_{0}\to \infty$, the following expression is obtained:

(27) $$G_{ij}\left(L,L_{0}\to \infty \right)=G_{ij}^{\infty }+\frac{\alpha_{ij}}{L}\;,$$

that describes the finite-size effects on the KBIs for a system in the grand canonical ensemble. Consistent with this limiting case in Equation (26), Equation (27) has been obtained from the thermodynamics of small systems [45,46].

For the investigation of Equation (26), we perform simulations for binary mixtures (A,B) of Lennard–Jones (LJ) fluids. We use a purely repulsive 12-6 LJ potential truncated and shifted with cutoff radius $2^{1/6}\sigma$. The potential parameters are chosen as $\sigma_{AA}=\sigma_{BB}=\sigma_{AB}=\sigma$, and $\epsilon_{AA}=1.2\epsilon$, $\epsilon_{BB}=1.0\epsilon$ with $\epsilon_{AB}=\left(\epsilon_{AA}+\epsilon_{BB}\right)/2=1.1\epsilon$. All the results are expressed in LJ units with energy ϵ, length σ, mass $m_{A}=m_{B}=m$, time $\sigma {\left(m/\epsilon \right)}^{1/2}$, temperature $\epsilon /k_{B}$ and pressure $\epsilon /\sigma^{3}$. As before, simulations are carried out using ESPResSo++ [32] with a time step of $\delta t/\left(\sigma {\left(m/\epsilon \right)}^{1/2}\right)=10^{-3}$. Constant temperature $k_{B}T=1.2\epsilon$ is enforced through a Langevin thermostat with damping coefficient $\gamma (\sigma {\left(m/\epsilon \right)}^{1/2})=1.0$. The size of the system is $N_{0}=23,328$ in the range of mole fractions of *A*-molecules $x_{A}=0.1,\cdots ,1.0$. The pressure is fixed at $P\sigma^{3}/\epsilon =9.8$ by adjusting the number density of the system at values around $\rho \sigma^{3}\approx 0.86$ (or $L_{0}/\sigma \approx 30$). We perform equilibration runs of $64\times 10^{6}$ MD steps and production runs of $2\times 10^{6}$ MD steps. To compute $G_{ij}\left(\lambda \right)$, we select 800 frames per trajectory and for each frame identify 1000 randomly-positioned subdomains with linear sizes ranging from $2<L/\sigma <L_{0}/\sigma$.

**Figure 10:**
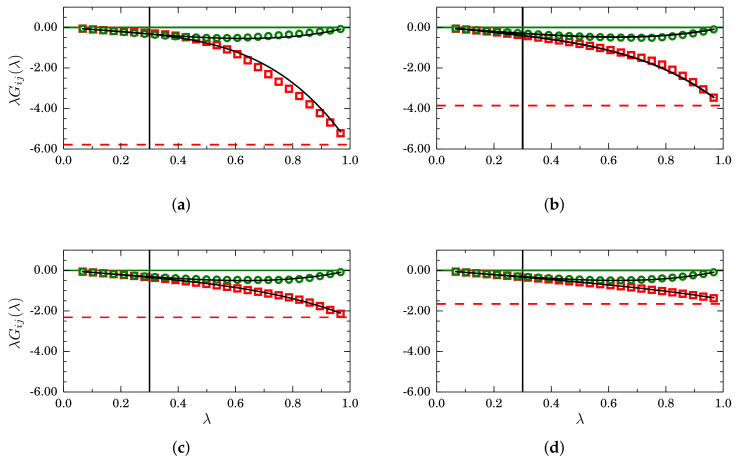
Scaled finite-size Kirkwood–Buff integrals $\lambda G_{ij}\left(\lambda \right)$ as a function of λ for different mole fractions: (**a**) $x_{A}=0.20$; (**b**) $x_{A}=0.30$; (**c**) $x_{A}=0.50$ and (**d**) $x_{A}=0.80$, for mixtures described by a TSLJ potential with $r_{c}/\sigma =2^{1/6}$. For clarity, only the cases $G_{AA}$ (red squares) and $G_{AB}$ (green circles) are plotted. In the asymptotic case λ→1, $G_{AB}\to 0$ and $G_{AA}\to 1/\rho_{A}$, as indicated by the horizontal green and red lines, respectively. The black curves correspond to Equation (26) with $G_{ij}^{\infty }$ and $\alpha_{ij}$ obtained from a simple regression analysis in the interval λ<0.3.

In Figure 10, results for finite-size KBIs are presented for four mole fractions, namely (a) $x_{A}=0.20$; (b) $x_{A}=0.30$; (c) $x_{A}=0.50$ and (d) $x_{A}=0.80$. Plots of $G_{AB}$ (green circles) tend to zero when λ→1, as indicated by the horizontal green lines. By contrast, $G_{AA}\to 1/\rho_{A}$ (indicated by horizontal red lines) when λ→1. The region λ<3, indicated by vertical black lines, is where simple linear regression is used to find $G_{ij}^{\infty }$ and $\alpha_{ij}$. By replacing such values in Equation (26), we obtained the black curves that, in all cases, superimpose on the simulation data for the full interval 0<λ<1.

The bulk KBIs are related to various thermodynamic quantities. For example, the isothermal compressibility is given by [39]:

(28) $$\kappa_{T}=\frac{1+\rho_{A}G_{AA}+\rho_{B}G_{BB}+\rho_{A}\rho_{B}\left(G_{AA}G_{BB}-G_{AB}^{2}\right)}{k_{B}T\left(\rho_{A}+\rho_{B}+\rho_{A}\rho_{B}\left(G_{AA}+G_{BB}-2G_{AB}\right)\right)}\;,$$

with $\rho_{A,B}$ the number density of the corresponding species.

**Figure 11:**
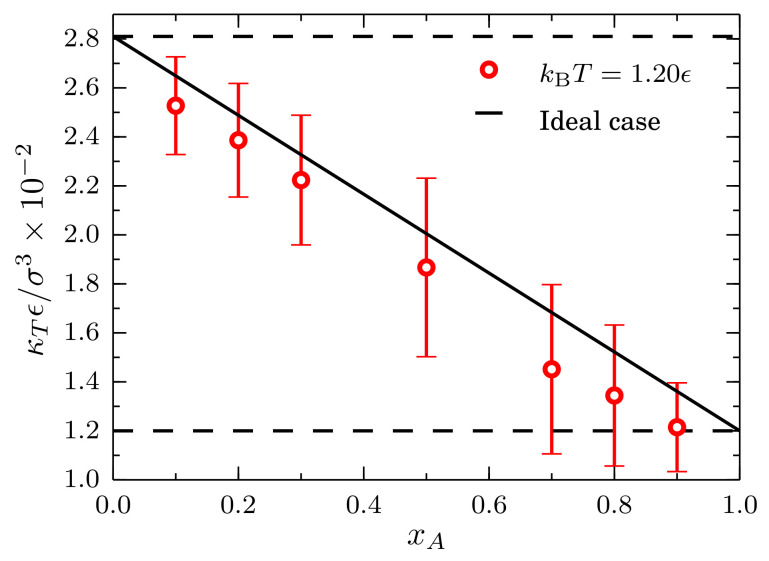
Isothermal compressibility at $k_{B}T=1.20\epsilon$ and $P\sigma^{3}/\epsilon =9.8$ as a function of the mole fraction of type-*A* particles $x_{A}$ for mixtures described by a TSLJ potential with $r_{c}/\sigma =2^{1/6}$. The horizontal black lines indicate the compressibility for a pure system of type-*A* particles $\kappa_{T}^{A}\epsilon /\sigma^{3}=0.012\left(1\right)$ and for a pure system of type-*B* particles $\kappa_{T}^{B}\epsilon /\sigma^{3}=0.0281\left(8\right)$. The red line is a guide to the eye. The ideal case corresponds to $\kappa_{T}=\left(1-x_{A}\right)\kappa_{T}^{B}+x_{A}\kappa_{T}^{A}$.

Results for the isothermal compressibility obtained from the $G_{ij}^{\infty }$ values are presented in Figure 11. Single component cases corresponding to systems composed by only type-*A* and type-*B* particles are indicated by the horizontal black lines. As expected, the system composed by strongly interacting particles, i.e., the type-*A*, has a lower compressibility. The behavior of the isothermal compressibility is nearly ideal since it follows closely the relation $\kappa_{T}=\left(1-x_{A}\right)\kappa_{T}^{B}+x_{A}\kappa_{T}^{A}$, with $\kappa_{T}^{A}\epsilon /\sigma^{3}=0.012\left(1\right)$ and $\kappa_{T}^{B}\epsilon /\sigma^{3}=0.0281\left(8\right)$, as indicated by the solid black line.

Finally, the extrapolated KBIs have been used to compute the derivative of the chemical potential of type-*A* particles with respect to the number density $\rho_{A}$ using the expression [39]:

(29) $$\frac{1}{k_{B}T}{\left(\frac{\partial \mu_{A}}{\partial \rho_{A}}\right)}_{P,T}=\frac{1}{\rho_{A}}+\frac{G_{AB}-G_{AA}}{1+\rho_{A}\left(G_{AA}-G_{AB}\right)}\;,$$

that, as has been done for the single component case, can be integrated to obtain [6]:

(30) $$\delta \mu_{A}=k_{B}T\int_{\rho_{A}^{0}}^{\rho_{A}}\left[\frac{1}{\rho_{A}^{\prime }}+\frac{G_{AB}-G_{AA}}{1+\rho_{A}^{\prime }\left(G_{AA}-G_{AB}\right)}\right]\;d\rho_{A}^{\prime }\;.$$

This is the chemical potential shifted by a reference chemical potential computed at density $\rho_{A}^{0}$ [4,43]. By removing the density and concentration terms of the chemical potential of an ideal mixture, the excess chemical potential can be written as:

(31) $$\delta \mu_{A}^{ex}=\delta \mu_{A}-k_{B}T\ln\left(x_{A}\right)-k_{B}T\ln\left(\rho_{A}\right)\;.$$

**Figure 12:**
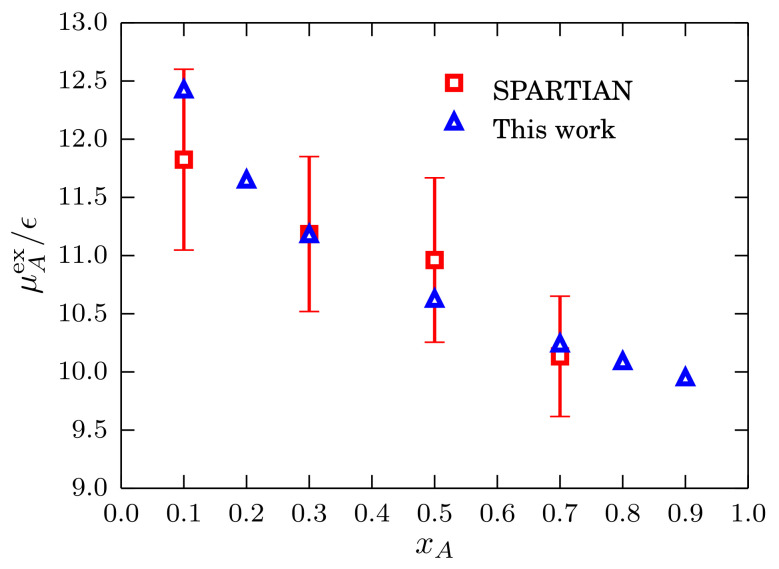
Excess chemical potential of type-*A* particles as a function of the mole fraction $x_{A}$ for mixtures described by a TSLJ potential with $r_{c}/\sigma =2^{1/6}$ at $k_{B}T=1.2\epsilon$ and $P\sigma^{3}/\epsilon =9.8$. Data points obtained with the method in [36], in particular for $x_{A}=0.3$, are used as a reference for the data points obtained with Equations (30) and (31).

We compare the results obtained using Equations (30) and (31) with the results obtained with the SPARTIAN method [36] and use the excess chemical potential result from $x_{A}=0.3$ to find the reference value. We present the results in Figure 12 where a good agreement between the two datasets is apparent. To conclude this section, it has been shown that the block analysis method constitutes a robust strategy to compute chemical potentials of liquids and mixtures in a wide range of density/concentration conditions.

## 5. Summary and Concluding Remarks

In general, a direct comparison between a real system and a finite-size simulation is prevented by the fixed and relatively small number of particles used in the latter. As has been encoded in the title of the paper, the spatial block analysis method employs a clever combination of finite-size effects, ensemble and boundary, and density fluctuations to extrapolate bulk isothermal compressibilities and Kirkwood–Buff integrals in the thermodynamic limit.

In this work, we have illustrated with prototypical Lennard–Jones liquids and liquid mixtures the working mechanisms of the method. Upon identifying the relevant finite-size effects and assessing their impact on the simulation results, we have intuitively introduced an analytical expression connecting the fluctuations measured in a small subdomain of the simulation box with the bulk isothermal compressibility for a single component fluid.

Subsequently, the same analytical expression has been rigorously obtained from a finite-size version of the Ornstein–Zernike integral equation. Using a challenging system close to critical point conditions, we have tested the range of validity of the method and obtained results in line with theoretical expectations.

Then, for a multicomponent system, we have applied the same protocol to the Kirkwood–Buff integrals. Using the corresponding analytical expression, it is possible to obtain the Kirkwood–Buff integrals in the thermodynamic limit. In both single and multicomponent systems, the method allows one to compute the chemical potential of a liquid/mixture for a wide range of density/concentration conditions, provided a single reference chemical potential has been determined using a different computational method. These results contribute to establishing the spatial block analysis method as a powerful tool to investigate systems where the accurate computation of the chemical potential is of paramount importance.

## Abbreviations

The following abbreviations are used in this manuscript:

SBA	Spatial block analysis
TSLJ	Truncated and shifted Lennard–Jones
MD	Molecular dynamics
TL	Thermodynamic limit
PBCs	Periodic boundary conditions
OZ	Ornstein–Zernike
KB	Kirkwood–Buff
